# Generation of Doubled Haploid Wheat-*Triticum urartu* Introgression Lines and Their Characterisation Using Chromosome-Specific KASP Markers

**DOI:** 10.3389/fpls.2021.643636

**Published:** 2021-05-13

**Authors:** Surbhi Grewal, Veronica Guwela, Claire Newell, Cai-yun Yang, Stephen Ashling, Duncan Scholefield, Stella Hubbart-Edwards, Amanda Burridge, Alex Stride, Ian P. King, Julie King

**Affiliations:** ^1^Nottingham BBSRC Wheat Research Centre, School of Biosciences, University of Nottingham, Loughborough, United Kingdom; ^2^Limagrain UK Limited, Bury St Edmunds, United Kingdom; ^3^School of Biological Sciences, University of Bristol, United Kingdom

**Keywords:** wheat, *Triticum urartu*, doubled haploids, introgressions, KASP markers, genotyping

## Abstract

Wheat is one of the most important food and protein sources in the world and although, in recent years wheat breeders have achieved yield gains, they are not sufficient to meet the demands of an ever-growing population. Development of high yielding wheat varieties, resilient to abiotic and biotic stress resulting from climate change, has been limited by wheat’s narrow genetic base. In contrast to wheat, the wild relatives of wheat provide a vast reservoir of genetic variation for most, if not all, agronomic traits. Previous studies by the authors have shown the transfer of genetic variation from *T. urartu* into bread wheat. However, before the introgression lines can be exploited for trait analysis, they are required to have stable transmission of the introgressions to the next generation. In this work, we describe the generation of 86 doubled haploid (DH) wheat-*T. urartu* introgression lines that carry homozygous introgressions which are stably inherited. The DH lines were characterised using the Axiom^®^ Wheat Relative Genotyping Array and 151 KASP markers to identify 65 unique *T. urartu* introgressions in a bread wheat background. DH production has helped accelerate the breeding process and facilitated the early release of homozygous wheat-*T. urartu* introgression lines. Together with the KASP markers, this valuable resource could greatly advance identification of beneficial alleles that can be used in wheat improvement.

## Introduction

*Triticum aestivum* (2*n* = 6*x* = 42; AABBDD) was formed via two sequential hybridisations. The first, between the A-genome donor *Triticum urartu* Thum ex. Gandil (2*n* = 2*x* = 14; A^u^A^u^) (Chapman et al., 1976; Dvořák et al., 1993) and the B-genome donor, a species similar to *Aegilops speltoides* 2*n* = 2*x* = 14; SS) (Sarkar and Stebbins, 1956; Riley and Chapman, 1958; Feldman et al., 1995) occurred approximately 0.36 to 0.5 million years ago (Huang et al., 2002; Dvořák and Akhunov, 2005; Marcussen et al., 2014) to form tetraploid wheat *Triticum turgidum ssp. dicoccoides* (2*n* = 4*x* = 28; AABB). The second hybridisation occurred either only once or twice about 8,000–10,000 years ago between allotetraploid wheat and *Aegilops tauschii*, the D-genome donor (2*n* = 2*x* = 14; DD) (Kihara, 1944; McFadden and Sears, 1944; Matsuoka, 2011). Thus, wheat is a relatively new species and in addition, due to its evolutionary pathway, has gone through a significant genetic bottleneck which has been further compounded by selection for traits of importance for its domestication (Charmet, 2011). It is therefore expected that hexaploid wheat carries significantly less genetic variation for exploitation in breeding programmes and research than its ancestral species and distant wild relatives, some of which evolved millions of years ago (Dempewolf et al., 2017). As a result, new sources of genetic variation for *T. aestivum* are invaluable for a wide range of traits.

The presence of the *Pairing Homoeologous 1* locus (*Ph1*) on the long arm of chromosome 5B of wheat (Okamoto, 1957; Riley and Chapman, 1958; Sears and Okamoto, 1958) restricts recombination at meiosis to homologous chromosomes (Sears, 1976). As a result, recombination cannot occur between the three related homoeologous genomes of wheat, i.e., the A, B and D genomes. The *Ph1* locus also prevents recombination between the chromosomes in wheat/wild relative interspecific F_1_ hybrids where the genome(s) of the wild relative is/are homoeologous to that/those of wheat, i.e., wild relatives that belong to the tertiary gene pool of wheat. In contrast, recombination can occur more freely between wild relatives and ancestral species that share a homologous genome in common with wheat, i.e., species such as *T. urartu* that carries the A-genome of wheat (Chapman et al., 1976).

*Triticum urartu* has not been extensively used to introduce new genetic variation into wheat although accessions have been identified with resistance to diseases such as stem rust (Rouse and Jin, 2011), powdery mildew (Qiu et al., 2005) and root lesion nematode (Sheedy et al., 2012) and other traits such as high net photosynthetic rate (Austin et al., 1982; Austin, 1986) and those that affect bread making quality (Martín et al., 2008; Cuesta et al., 2015).

The transfer of chromosome segments or introgressions from wild relatives and ancestral species into wheat has been hindered in the past through the inability to know exactly what had been transferred. Much of the work was centred on the transfer of genetic variation for a trait of interest and relied very heavily on phenotyping for that trait in order to track the transfer. The molecular tools that are now available, however, enable the identification and characterisation of introgressions and the tracking of them through the different generations in a crossing programme.

The use of the new technologies at the Nottingham BBSRC Wheat Research Centre (WRC) has allowed the transfer of hundreds of introgressions from a variety of wild relatives into wheat, e.g., *Amblyopyrum muticum* (King et al., 2017), *Thinopyrum bessarabicum* (Grewal et al., 2018b), *Ae. speltoides* (King et al., 2018), *T. urartu* (Grewal et al., 2018a), *Triticum timopheevii* (Devi et al., 2019), *Thinopyrum elongatum* (Baker et al., 2020), and *Aegilops caudata* (Grewal et al., 2020b). However, without identifying what genetic variation is carried by the introgressions, the lines produced will remain quite simply as ‘seeds in a packet’ of unknown agronomic potential. It is therefore essential that these introgressions are phenotyped as extensively as possible for as many different traits as possible.

In order that these lines can be analysed in a range of environments throughout the world they need first be multiplied and then distributed to collaborators. In order to multiply the introgression lines, however, they must first be homozygous to ensure that they are stably inherited at each generation during multiplication. This paper describes the exploitation of the doubled haploid (DH) procedure to generate homozygous introgressions of *T. urartu* from heterozygous introgressions and their characterisation via molecular markers and genomic *in situ* hybridisation (GISH).

## Materials and Methods

### Plant Materials

A selection of the introgression lines generated and described by Grewal et al. (2018a) were used for DH production. In summary, *T. aestivum* cv. Chinese Spring *ph1* mutant was pollinated with *T. urartu* [accessions 1010001, 1010002, and 1010006 obtained from the Germplasm Resources Unit (GRU) at John Innes Centre, United Kingdom]. The F_1_ interspecific hybrids produced were backcrossed to *T. aestivum* cv. Paragon, carrying the wild-type *Ph1* locus, to obtain BC_1_ progeny which were then recurrently backcrossed with Paragon to produce a BC_3_ population. Selected BC_3_ plants were crossed to maize to initiate DH production. When this work was initially undertaken, no suitable SNP genotyping platform was available to enable marker-assisted selection and backcrossing. Thus, there was no indication of which BC_3_ lines carried *T. urartu* introgressions and in the absence of GISH capability to detect *T. urartu* derived A-genome segments, the BC_3_ plants were chosen at random for DH production.

### Doubled Haploid Production

The DH production procedure used was as described by Laurie and Reymondie (1991) and used previously to generate wheat-*Am. muticum* DH lines (King et al., 2019). In summary, wheat spikes were emasculated and then pollinated with maize (cultivars Northern Extra Sweet, Prelude and Sundance). One day after pollination, internodes below pollinated spikes were filled with 10 mg l^–1^ of 2,4-dichlorophenoxyacetic acid (2,4-D solution) with a syringe and the holes sealed with petroleum jelly. The 2,4-D solution was also injected into each floret. After 14–21 days immature seeds were harvested, and haploid embryos were excised and cultured under sterile conditions. Subsequent colchicine treatment to obtain DH seedlings was carried out as described in Nemeth et al. (2015). Plants were grown to maturity in soil and self-fertilised seed was harvested.

### Development of KASP Markers

Grewal et al. (2020a) generated 2374 SNPs between hexaploid wheat and ten wild relative species and converted 1000 of these SNPs into KASP assays. Of the remaining SNPs, 79 were selected (as set 1), that were polymorphic between wheat and *T. urartu* and *Am. muticum* for conversion to chromosome-specific KASP assays as described by Grewal et al. (2020a) and designated codes between WRC1001-WRC1079 (Supplementary Table 2).

A list of 1742 SNPs was also combined from a core set of 960 SNPs already used to develop a KASP wheat panel by University of Bristol for LGC, Biosearch Technologies^1^ (last accessed on 11 June 2020) and a set of 782 SNPs that were identified as reliable for use in wheat via various genotyping platforms including as KASP markers (Burridge et al., 2018). After removing duplicates, 1572 SNPs were obtained which were all available as probes on the 820K Axiom^®^ Array (Winfield et al., 2016). Through analysis of the KASP primers dataset of all the 820K Axiom^®^ SNPs, generated using PolyMarker (Ramirez-Gonzalez et al., 2015) and available at CerealsDB^2^ (last accessed 11 June 2020), information on significant BLAST hits for each of these SNPs was obtained. The 229 SNPs that were observed to be present on a single contig in the wheat genome (Refseqv1.0) (IWGSC et al., 2018) were selected (as set 2). Pre-validated KASP assay primers for these SNPs were obtained via CerealsDB and designated codes between WRC1080-WRC1308 (Supplementary Table 2). A further 77 chromosome-specific KASP assays were also selected (as set 3) that were simultaneously developed from the 820K Axiom^®^ Array to be polymorphic between wheat and *Th. bessarabicum*. These were designated codes WRC1317-WRC1393 (Supplementary Table 2).

### Genotyping of Wheat-*T. urartu* DH Lines

All the DH lines were initially genotyped with the Axiom^®^ 36K Wheat-Relative Genotyping Array (Thermo Fisher Scientific, United Kingdom) (Winfield et al., 2016; King et al., 2017) and subsequently genotyped with KASP markers (LGC, Biosearch Technologies, United Kingdom). The latter included the three sets of KASP assays as described above in addition to chromosome-specific KASP markers reported by Grewal et al. (2020a).

Genomic DNA was isolated from leaf tissue of 10-day-old seedlings in a 96-well plate as described by Thomson and Henry (1995). All DH lines were genotyped alongside four wheat genotypes (Chinese Spring, Paragon, Pavon, and Highbury) and the *T. urartu* accessions as controls.

Genotyping with the Axiom^®^ 36K Wheat-Relative Array was performed by including all the DH lines on the array with other wheat-*T. urartu* backcrossed populations. Genotypes were called, using the methodology described by Grewal et al. (2018a), for a subset of probes present on the genetic map of *T. urartu* as reported by the same study.

All DH lines shown to have an introgression from *T. urartu* in the array genotyping were subsequently genotyped with KASP markers after a round(s) of self-fertilisation. The genotyping procedure was as described by Grewal et al. (2020b). In summary, the genotyping reactions were set up using the automated PIPETMAX^®^ 268 (Gilson, United Kingdom) and performed in a ProFlex PCR system (Applied Biosystems by Life Technology) in a final volume of 5 μl with 1 ng genomic DNA, 2.5 μl KASP reaction mix (ROX), 0.068 μl primer mix and 2.43 μl nuclease free water. PCR conditions were set as 15 min at 94°C; 10 touchdown cycles of 10 s at 94°C, 1 min at 65–57°C (dropping 0.8°C per cycle); and 35 cycles of 10 s at 94°C, 1 min at 57°C. Fluorescence detection of the reactions was performed using a QuantStudio 5 (Applied Biosystems) and the data analysed using the QuantStudio^TM^ Design and Analysis Software V1.5.0 (Applied Biosystems).

**FIGURE 1:**
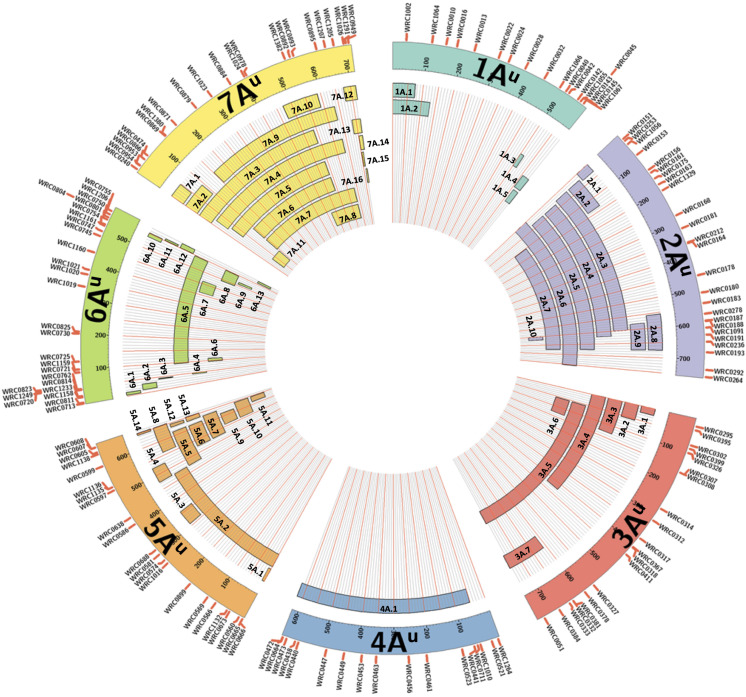
A graphical representation of the sizes of the 65 unique introgressions of *T. urartu*, along each *T. urartu* chromosome, found in the DH lines. In each of the seven chromosomes, the KASP markers are shown ordered according to their physical position (Ling et al., 2018). Each segment is given a unique name which is used to characterise the DH lines in Table 2.

All KASP markers included in the final list of working assays were used in a BLAST analysis against the wheat genome sequence Refseqv1 (IWGSC et al., 2018) and the *T. urartu* genome sequence (Ling et al., 2018). The markers within a linkage group were ordered according to their physical positions on the *T. urartu* genome (Supplementary Table 3) and used to represent the approximate sizes of the chromosome segments introgressed from *T. urartu* into wheat (Figure 1).

### Genomic *in situ* Hybridisation (GISH)

The protocol for genomic *in situ* hybridization (GISH) and preparation of chromosome spreads was as described in Kato et al. (2004), King et al. (2017), and Grewal et al. (2020b). In summary, genomic DNA was isolated from the three putative diploid progenitors of bread wheat, i.e., *T. urartu* (A genome), *Ae. speltoides* (B genome), and *Ae. tauschii* (D genome), using extraction buffer [0.1 M Tris-HCl (pH 7.5), 0.05 M EDTA (pH 8.0), 1.25% SDS]. Samples were incubated at 65°C for 1 h before being placed on ice and mixed with ice cold 6 M NH_4_C_2_H_3_O for 15 min. The samples were then spun down, the supernatant mixed with isopropanol to pellet the DNA and the isolated DNA further purified with phenol/chloroform and resuspended in 2 × SSC and 1 × TE buffer, pH 7.0. The genomic DNA of (1) *T. urartu* was labelled by nick translation with ChromaTide^TM^ Alexa Fluor^TM^ 488-5-dUTP (Invitrogen; C11397; coloured green), (2) *Ae. speltoides* was labelled by nick translation with DEAC-dUTP (Jena Bioscience; NU-803-DEAC; coloured bluish purple), and (3) *Ae. tauschii* was labelled with ChromaTide^TM^ Alexa Fluor^TM^ 594-5-dUTP (Invitrogen; C11400; coloured red).

Slides were probed using a probe mixture that contained the labelled genomic DNAs of *T. urartu*, *Ae. speltoides* and *Ae. tauschii* in the ratio 3:3:4, respectively, in 2 × SSC and 1 × TE buffer, pH 7.0, in a final volume of 10 μl per slide. Slides were counterstained with Vectashield mounting medium with 4′-6-diamidino-2-phenylindole dihydrochloride (DAPI) and analysed using a Zeiss Axio ImagerZ2 upright epifluorescence microscope (Carl Zeiss Ltd., Oberkochen, Germany) with filters for DAPI (blue), Alexa Fluor 488 (green), Alexa Fluor 594 (red), Alexa Fluor 546 and DEAC (aqua). Photographs were taken using a MetaSystems Coolcube 1 m CCD camera together with the Metafer v.4 software (MetaSystems GmbH, Altlussheim, Germany) and further image analysis was carried out using ISIS v.5.8.5 (MetaSystems GmbH, Altlussheim, Germany).

## Results

### Generation of Wheat-*T. urartu* DH Lines

Forty-nine BC_3_ plants were pollinated with maize in order to generate DH lines. In total, 183 DH seedlings were obtained from 26 BC_3_ plants (Table 1). Of these, 127 (69%) DH plants grew to maturity. A further 13 DH plants were found to be sterile resulting in the generation of 114 fertile DH plants (Table 1) that set F_1_ seed. Out of the 49 BC_3_ plants used for DH production, 20 (41%) eventually produced fertile DH plants.

**TABLE 1:** Number of BC_3_ plants (and their original code) involved in the various stages of DH production including the number of DH plants that were fertile and carried *T. urartu* segments, their DH line codes and details of which *T. urartu* accession was present in each DH line.

Tu* accession	Original BC_3_ plant code	Number of embryos harvested	Number of DH plants grown to maturity	Number of sterile DH plants	Number of DH plants without Tu segments	Number of plants with Tu segments and producing seed	Line codes for DH plants with Tu segments and producing seed
1010002	BC3-203B	7	7	0	1	6	208–210; 212–214
	BC3-203C	19	15	0	10	5	215; 219; 222; 226; 229
	BC3-203D	7	4	1	1	2	232; 336
	BC3-203E	31	26	0	11	15	233–235; 240; 242–244; 247–249; 253–255; 257; 259
1010006	BC3-204C	1	1	–	1	0	–
	BC3-204D	12	8	2	2	4	261; 263; 266; 268
1010006	BC3-205A	1	1	1	0	0	–
	BC3-205B	7	1	0	0	1	270
	BC3-205D	1	0	–	–	–	–
1010002	BC3-206A	1	0	–	–	–	–
	BC3-206B	2	0	–	–	–	–
	BC3-206C	6	1	0	0	1	271
	BC3-206D	6	5	0	0	5	272–276
1010001	BC3-208E	2	1	0	0	1	277
1010001	BC3-209B	4	3	2	0	1	278
	BC3-209D	1	0	–	–	–	–
1010006	BC3-210A	3	3	1	0	2	281–282
	BC3-210B	7	5	0	0	5	283–286; 353
	BC3-210C	7	4	2	1	1	288
	BC3-210E	14	13	0	1	12	289; 291–301
1010002	BC3-211A	1	0	–	–	–	–
	BC3-211D	10	4	0	0	4	302–305
1010002	BC3-212B	11	10	0	0	10	306–315
	BC3-212C	1	1	0	0	1	316
	BC3-212D	9	7	3	0	4	317–318; 320–321
	BC3-212E	12	7	1	0	6	324–329
**Total**		**183**	**127**	**13**	**28**	**86**	

### Molecular Characterisation With the Axiom^®^ Wheat-Relative Genotyping Array

The 114 fertile DH plants were genotyped using the Axiom^®^ Wheat Relative Array. However, genotypes were called for a subset of the probes, 368 of those that were present on the genetic map of *T. urartu* (Grewal et al., 2018a). The resulting marker analysis indicated that 28 (25%) DH plants did not carry any *T. urartu* chromosomes and/or wheat-*T. urartu* segments. Thus, in total, 86 wheat-*T. urartu* fertile DH lines carrying *T. urartu* introgressions were obtained in this study (Table 1). The linkage group(s) of the *T. urartu* introgression(s) present in each of these 86 DH lines, as obtained through array genotyping, is shown in Table 2.

**TABLE 2:** List of all 86 wheat-*T. urartu* DH lines showing the linkage groups detected in each line via the Axiom array and KASP markers and the name of the unique introgression (corresponding to Figure 1) and its potential location in the wheat genome.

DH line number(s)	LG of Tu segment(s) from array genotyping	LG of Tu segment(s) from KASP genotyping (number of segments from that LG when more than 1)	Name of segment(s) corresponding to Figure 1, identified through KASP genotyping	Wheat chromosome potentially present in the recombinant
208	3, 6, 7	3, 5, 6, 7	3A.2, 5A.11, 6A.4, 7A.7	3A, 5A, 6A, 7A
209	2, 5, 6	2, 5(2), 6	2A.3, 5A.4, 5A.10, 6A.4	2A, 5A, 5A, 6A
210	2, 3, 5, 7	2, 3, 5(2), 7	2A.3, 3A.7, 5A.4, 5A.9, 7A.11	2A, 3A, 5A, 5A, 7A
212	2, 5	2	2A.2	2A
213	3, 5	1, 3, 5	1A.2, 3A.1, 5A.12	1A, 3A, 5A
214	2, 3, 5, 6	2, 3, 5(2), 6	2A.3, 3A.3, 5A.4, 5A.9, 6A.4	2A, 3A, 5A, 5A, 6A
215; 219; 222; 226; 229	6, 7	6, 7	6A.4, 7A.8	6A, 7A
232	3, 5, 7	3, 5, 7	3A.2, 5A.13, 7A.12	3A, 5A, 7A
336	3, 5, 7	3, 5(2), 7	3A.2, 5A.4, 5A.13, 7A.12	3A, 5A, 5A,7A
233	5, 7	5, 7	5A.8, 7A.14	5D, 7B
234; 253	5	5 (2)	5A.13, 5A.13,	5A, 5D
235; 247	5	5, 7	5A.13, 7A.12	5D, 7A
240	5	5	5A.8	5D
242; 244	5, 7	5(3), 7	5A.4, 5A.8, 5A.13, 7A.14	5A, 5A, 5D, 7B
243	5, 7	5(3), 7(2)	5A.4, 5A.8, 5A.13, 7A.12, 7A.14	5A, 5A, 7A, 7B
248; 254	5	5(3)	5A.4, 5A.13, 5A.13	5A, 5A, 5D
249; 257	5	5(2), 7	5A.4, 5A.8, 7A.12	5A, 5A, 7A
255	5	5(2), 7	5A.4, 5A.8, 7A.12	5A, 5D, 7A
259	5, 7	5, 7(2)	5A.13, 7A.12, 7A.14	5D, 7A, 7B
261	3	3	3A.1	3A
263	6, 7	6, 7	6A.5, 7A.9	6A, 7A
266	6, 7	5, 6, 7	5A.13, 6A.5, 7A.9	5A, 6A, 7A
268	3, 7	3, 7	3A.1, 7A.9	3A, 7A
270	5, 6	5, 6	5A.2, 6A.2	5A, 6A, 7A
271	3, 5	3(2), 5	3A.4, 3A.5, 5A.7	3D, 3D, 5A
272	3, 7	1, 3(2), 7	1A.3, 3A.4, 3A.7, 7A.2	1A, 3A, 3A, 7A
273	3	3(2)	3A.4, 3A.5	3D, 3D
274	3, 7	3, 7	3A.1, 7A.4	3A, 7A
275	3, 7	1, 3, 7	1A.3, 3A.5, 7A.4	1A, 3D, 7A
276	3, 7	3(2), 7	3A.4, 3A.5, 6A.4, 7A.2	3D, 3D, 6A, 7A
277	1, 6	1, 2, 6(3)	1A.5, 2A.10, 6A.6, 6A.7, 6A.9	1A, 2A, 6A, 6A, 6A
278	1, 2, 6	1, 2, 6	1A.4, 2A.1, 6A.12	1*, 2A, 6A
281	3, 7	3, 7(2)	3A.7, 7A.2, 7A.15	3A, 7A, 7B
282	3, 4, 7	3, 4, 7(2)	3A.7, 4A.1, 7A.11, 7A.15	3A, 4D, 7A, 7A
283	3	3	3A.7	3A
284	5	5	5A.9	5A
285	2	2	2A.4	2A
286	2, 5, 7	2, 5, 7	2A.4, 5A.5, 7A.5	2A, 5A, 7A
353	3, 5, 7	3, 5, 7	3A.7, 5A.4, 7A.3	3A, 5A, 7A
288	4, 6	4, 6	4A.1, 6A.4	4D, 6A
289	2, 5, 7	2, 5, 7	2A.4, 5A.6, 7A.6	2A, 5A, 7A
291	2, 5	2, 5(2)	2A.4, 5A.6, 5A.13	2A, 5A, 7A
292	2	2, 5, 7	2A.4, 5A.14, 7A.15	2A, 5A, 7B
293	2, 5	2, 5, 7	2A.4, 5A.6, 7A.15	2A, 5A, 7B
294	2, 7	2, 7(2)	2A.4, 7A.6, 7A. 15	2A, 7A, 7B
295	7	5, 7(2)	5A.13, 7A.6, 7A.15	5A, 7A, 7B
296	2, 5, 7	2, 5(2), 7	2A.4, 5A.6, 5A.13, 7A.6	2A, 5A, 5A, 7A
297	2, 5, 7	2, 5, 7(2)	2A.4, 5A.13, 7A.6, 7A.15	2A, 5A, 7A, 7B
298	5	5(2)	5A.6, 5A.13	5A, 5A
299	5, 7	5(2), 7(2)	5A.6, 5A.12, 7A.6, 7A.15	5A, 5A, 7A, 7B
300	2, 5, 7	2, 5, 7(2)	2A.4, 5A.6, 7A.6, 7A.15	2A, 5A, 7A, 7B
301	2, 7	2, 7	2A.4, 7A.6	2A, 7A
302	5	–	–	–
303: 305	7	7	7A.10	7A
304	2, 5	2	2A.1	2A
306	1, 2	1, 2, 7	1A.2, 2A.5, 7A.13	1A, 2A, 7A
307	1, 5	1, 5, 7	1A.2, 5A.4, 7A.13	1A, 5A, 7A
308; 310	5	5	5A.4	5A
309; 312	1, 5	1, 5	1A.2, 5A.4	1A, 5A
311	1, 2, 5	1, 2, 5, 7	1A.2, 2A.5, 5A.4, 7A.13	1A, 2A, 5A, 7A
313	1	1	1A.2	1A
314	1, 2, 5	1, 2, 5	1A.2, 2A.5, 5A.4	1A, 2A, 5A
315	2	1, 2	1A.2, 2A.5	1A, 2A
316	2, 5, 6	2, 5, 6	2A.9, 5A.4, 6A.8	2A, 5A, 6A
317	5	5(3)	5A.1, 5A.3, 5A.4	5D, 5D, 5A
318	2, 5	2, 5	2A.7, 5A.4	2A, 5A
320	5	5	5A.4	5A
321	1, 2, 5	1, 2, 5(3)	1A.1, 2A.8, 5A.1, 5A.3, 5A.4	1A, 2A, 5D, 5D, 5A
324; 325, 329	2	2	2A.6	Whole
326; 328	5	–	–	–
327	2, 5	2	2A.6	Whole

*Wheat subgenome cannot be confirmed through KASP genotyping.

### Development of KASP Markers

The progeny of the 86 DH lines were further genotyped with KASP markers to validate the results of the array genotyping and identify the wheat chromosome(s) involved in the recombinant chromosomes.

Grewal et al. (2020a) produced a panel of 710 KASP markers validated across ten wild relative species of which 118 were polymorphic between bread wheat and *T. urartu*. This subset of markers, consisting of 115 chromosome-specific and 3 chromosome-nonspecific KASP assays (Table 3), were used to genotype the DH lines in the present study. All 118 markers were able to distinguish between wheat and *T. urartu* alleles and were thus able to detect a vast majority of the introgressions in the DH plants. However, there were still gaps in the marker set where the array genotyping had detected segments but no KASP markers were available.

**TABLE 3:** Number of chromosome-specific and nonspecific KASP markers developed for each wheat chromosome in the current study (from sets 1, 2, and 3) versus previously developed KASP markers.

	Chromosome-specific KASP markers		Chromosome-nonspecific KASP numbers		
Wheat chromosome	From Grewal et al. (2020a)	From sets 1, 2, and 3	From Grewal et al. (2020a)	From sets 1, 2, and 3	Subtotal
1A	10	1	0	1	12
1B	1	0	0	2	3
1D	3	1	0	0	4
2A	16	2	0	1	19
2B	3	0	0	0	3
2D	3	0	1	0	4
3A	12	0	0	0	12
3B	3	0	1	0	4
3D	2	0	1	0	3
4A	11	1	0	0	12
4B	2	0	0	1	3
4D	2	0	0	0	2
5A	11	5	0	0	16
5B	1	0	0	0	1
5D	5	0	0	0	5
6A	10	10	0	0	20
6B	3	0	0	0	3
6D	4	0	0	0	4
7A	8	6	0	0	14
7B	2	1	0	1	4
7D	3	0	0	0	3
**Total**	**115**	**27**	**3**	**6**	**151**

To achieve better detection of all *T. urartu* introgressions with KASP markers, a further three sets of KASP assays which were being developed in tandem with this work were used for genotyping the DH lines. From set 1, 13 (16%) out of the 79 assays failed to amplify the templates and produced no genotypes. Of the working assays (those that detected at least two wheat genotypes), 14 KASP markers were validated as being polymorphic between wheat and *T. urartu*. Ten of these KASP markers were chromosome-specific in wheat. From set two, 209 (90%) KASP assays amplified the control samples, of which 16 markers were found to be polymorphic for *T. urartu* with 14 of these being chromosome-specific in wheat. Eighteen (23%) out of the 77 assays failed from set 3 and 3 KASP markers were identified as being polymorphic for *T. urartu* from amongst the working assays. All three assays were chromosome specific in wheat. In summary, out of the 385 KASP assays designed to work across various wild relatives and wheat genotypes in the three sets, 334 (∼87%) were found to be polymorphic between wheat and various other wild relatives (data not shown) and of those 33 (∼10%) were found to work for *T. urartu* in a wheat background. Of these new KASP assays for *T. urartu*, 27 were chromosome-specific in wheat and 6 were chromosome-nonspecific (Table 3). Combining these with those previously developed by Grewal et al. (2020a) resulted in a total of 151 KASP markers that were used to characterise the wheat-*T. urartu* DH lines in this study (Table 3 and Figure 1).

### Genotyping of DH Lines With Chromosome-Specific KASP Markers

Through KASP genotyping with chromosome-specific assays, we were able to detect 216 *Triticum urartu* introgressions across the 86 DH lines with 65 unique introgressions (segments or whole chromosomes) that were present in one or more DH lines (Table 2). The KASP markers were able to detect the presence of one (or more) additional *T. urartu* linkage group (LG) in 19 of the DH lines as compared to the Axiom array. Although, the reverse was true for six DH lines indicating that there were still significant gaps in the detection capacity of the KASP markers.

The introgressions were named after the *T. urartu* LG they were derived from and Table 2 provides details of which LG(s) and corresponding introgressions, as detected by KASP markers, are present in each DH line. In addition, Figure 1 shows the relative size of each of these introgressions as compared to the size of the *T. urartu* chromosomes (Ling et al., 2018). In total, we obtained 5 introgressed segments from LG 1, 9 segments and a pair of whole chromosomes from LG 2, 7 segments from LG3, only one large segment from LG4, 14 mostly small segments from LG 5, 13 segments from LG 6 and 15 segments of varying sizes from LG 7 of *T. urartu* in the wheat background. The maximum number of *T. urartu* introgressions in a DH line was five with DH lines 210, 214, 243, 277, and 321 all having five introgressions each (Table 2).

Genotyping of homozygous introgression lines with chromosome-specific KASP markers also allows the potential identification of the wheat chromosome recombined with the *T. urartu* segment (Grewal et al., 2020a,b) due to the absence of the wheat-specific allele in homozygous lines which will instead only show the presence of the wild-relative-specific allele for a marker that lies within the introgression. Since the lines characterised in this work are DH lines, they are expected to be homozygous for every introgression from *T. urartu* and thus, the chromosome-specific KASP markers should be able to identify the wheat chromosome recipient of the introgression.

Of the 65 introgressions, the A-genome specific KASP markers indicated that 54 were with an A-genome chromosome of wheat (Table 2) where the genotypes were called as homozygous for the wild relative alleles. During GISH analysis, the genomic probe used for detecting A-genome chromosomes in wheat is prepared from *T. urartu* genomic DNA and expectedly also detects A^u^ chromosome segments of *T. urartu* in a wheat background. Thus, it is not possible to validate the presence and size of the *T. urartu* segments introgressed into the A genome of wheat using GISH.

Of the remaining 11 introgressions, the chromosome-specific KASP markers indicated that introgressions 5A.1 and 5A.3 were with Chr 5D of wheat and 7A.14 and 7A.15 were with Chr 7B of wheat (Table 2). However, all of these were potentially too small to be visualised using GISH. Similarly, 1A.4 was potentially too small to be detected by GISH and the markers were also unable to conclusively indicate which subgenome of wheat it had recombined with. Where the introgressions had not directly recombined with the A genome of wheat and were larger segments, GISH analysis was used to validate the KASP genotyping results.

### GISH Analysis of Wheat-*T. urartu* DH Lines

**FIGURE 2:**
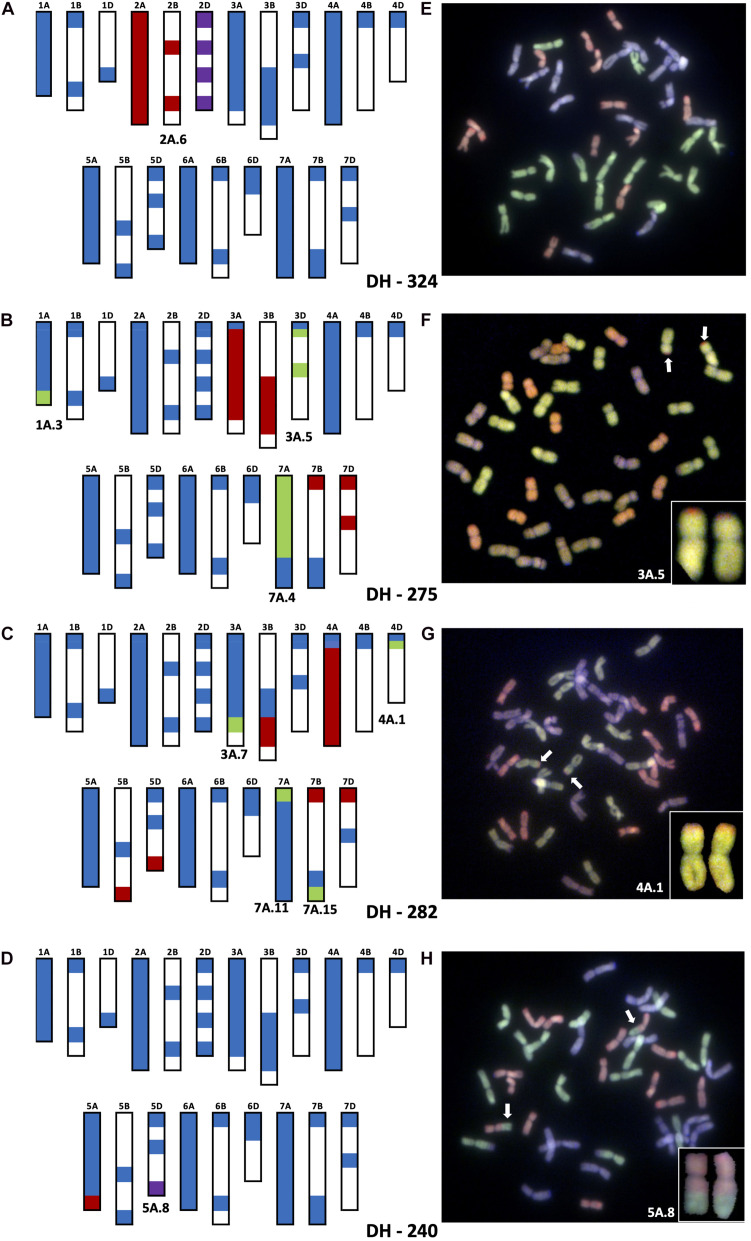
Molecular characterization of wheat–*T. urartu* DH lines showing genotyping analysis with chromosome–specific KASP assays **(A–D)** and Genomic *in situ* hybridization (GISH) images of root metaphase spreads **(E–H)**. Panels **(A,E)** showing presence of whole chromosome 2Au (2A.6) substituting for Chr 2D in DH-324. Panels **(B,F)** showing the introgression of segment 3A.5 into Chr 3D of wheat in DH-275. Panels **(C,G)** showing the introgressions of segment 4A.1 into Chr 4D of wheat in DH-282. Panels **(D,H)** showing the introgression of segment 5A.8 into Chr 5D of wheat. In the genotyping data, all heterozygous calls are shown in red, homozygous wild relative calls in green, homozygous wheat calls in blue and null calls are shown in purple. White spaces indicate regions where KASP markers are not present. For the GISH, green represents the A-genome of wheat or A^u^-genome of *T. urartu*, greyish purple, the B-genome and red, the D-genome of wheat. Identifiable introgressions are indicated by white arrows.

Introgression 2A.6 was found to be a pair of whole 2A^u^ chromosomes that replaced a pair of wheat 2D chromosomes in DH lines 324, 325, 327 and 329 making these 2A^u^(2D) substitution lines. Figure 2A shows the genotyping of DH 324 with KASP markers and validation of the result with GISH. The heterozygous calls for markers on Chr 2A and 2B of wheat indicate the presence of a very large segment from Chr 2A^u^ of *T. urartu* but the lack of any homozygous calls indicates that the introgression has not recombined with a wheat chromosome. Instead, the null calls for markers on 2D indicate that the pair of 2D chromosomes is missing in this line. GISH analysis confirms the presence of 16 A genome chromosomes (the probe for the wheat A-genome chromosomes detects any A^u^ genome chromosomes with the same colour) and 12 D-genome chromosomes thereby indicating that introgression 2A.6 is potentially a pair of Chr 2A^u^.

The markers indicated that introgressions 3A.4 and 3A.5 had recombined with the D-genome of wheat and in particular chromosome 3D. Both introgressions are present in DH lines 271, 272 and 276, while DH 275 only contained segment 3A.5 (Table 2 and Figure 2B). The presence of 3A.5 in Chr 3D of DH 275 is indicated by the presence of heterozygous calls for markers on Chr 3A and Chr 3B but homozygous calls (for the *T. urartu* allele) for markers on Chr 3D (except those on the distal end of the short arm on Chr 3A and 3D which gave homozygous calls for the wheat allele) as shown in Figure 2B. This was validated by GISH analysis that showed a pair of recombinant chromosomes with a large A-genome or equivalent segment (likely Chr 3A^u^ of *T. urartu*) and a very small segment of the D genome of wheat on the distal end of the short arm (Figure 2B). DH 275 also has introgressions 1A.3 and 7A.4 but since the markers indicate they have gone into Chr 1A and Chr 7A of wheat, respectively (through homozygous *T. urartu* calls for markers on these chromosomes), they cannot be observed through GISH.

In DH lines 282 and 288, introgression 4A.1 had recombined with Chr 4D of wheat (Table 2) as indicated by marker analysis in Figure 2C. The KASP markers on the very distal ends of the short arms of chromosomes 4A, 4B and 4D of wheat gave homozygous wheat calls but the majority of the markers on Chr 4A gave heterozygous calls while one marker on Chr 4D gave a homozygous call for the *T. urartu* allele indicating that except for a very small region at the distal end of the short arm, the majority of Chr 4D had been potentially replaced with Chr 4A^u^ of wheat. The markers on the very distal ends of the long arms of Chr 5B and 5D also give heterozygous calls due to the 4AL-5AL translocation present in *T. urartu* and in wheat (Liu et al., 1992; King et al., 1994; Devos et al., 1995; Dvořák et al., 2018). Thus, since 4A.1 has a translocated segment from Chr 5A^u^L which is homoeologous to the corresponding regions in Chr 5BL and Chr 5DL, the markers on the latter two chromosomes also detect this segment (Figure 2C). This was validated by GISH analysis that showed a pair of recombinant chromosomes with a large A-genome or equivalent segment (likely Chr 4A^u^ of *T. urartu*) and a very small segment of the D genome of wheat on the distal end of the short arm (Figure 2C). DH 282 also had introgressions 3A.7 and 7A.11 that recombined with Chr 3A and Chr 7A of wheat, respectively, as indicated by the markers in addition to a very small introgression 7A.15 which had potentially gone into Chr 7B of wheat (Table 2 and Figure 2C).

The markers also showed that introgressions 5A.8 and 5A.13 had recombined with both Chr 5A and Chr 5D of wheat in various DH lines. A number of combinations of these introgressions were found in DH lines 232–259 (Table 2). Figure 2D shows the genotype of DH 240 which was shown to have 5A.8 in Chr 5D. The heterozygous calls on the distal end of Chr 5AL indicated the presence of a *T. urartu* Chr 5A^u^L segment which is really translocation of Chr4A^u^L. The null call for the marker at the distal end of Chr 5D indicated that this segment had replaced the corresponding region of Chr 5D in this DH line (Figure 2D).

Root metaphase spreads were obtained for a total of 75 out of the 86 DH lines for GISH analysis. In addition to observing recombinant chromosomes, GISH also allowed the detection of all the wheat chromosomes. Even though no GISH images were obtained for DH lines 265 and 271, the markers suggested that these lines were missing a pair of 1D and 5D chromosomes, respectively. Supplementary Table 1 provides details of the number of chromosomes observed for each subgenome in wheat for all DH lines with GISH images.

## Discussion

The primary aim of the work carried out at the Nottingham BBSRC Wheat Research Centre is to generate stable homozygous wheat-wild relative introgressions lines that can be distributed to the wider wheat community for trait discovery. In this work, we describe a strategy to generate large numbers of stable introgressions in wheat from *T. urartu* using the DH method on some of the lines from the back-crossed population generated by Grewal et al. (2018a) and molecular characterisation of these lines. This is similar to the work reported by King et al. (2019) where DHs were generated for wheat-*Am. muticum* introgression lines and genotyped using the Axiom^®^ Wheat Relative Genotyping Array. However, the wheat-*T. urartu* DH introgression lines produced in this study have been characterised with chromosome-specific KASP markers in addition to the Axiom^®^ Array. KASP markers were found to be more flexible and cost-effective as a genotyping platform and allowed, in most cases, to identification of wheat chromosomes into which the *T. urartu* segments had introgressed.

### Production of Stable Homozygous Wheat-*T. urartu* DH Lines

From the 49 BC_3_ plants that were used to generate the DH population, a total of 114 fertile DH plants were generated from 20 of them (41%) with the array genotyping indicating that 86 of the DH lines carried a *T. urartu* segment or whole chromosomes (Tables 1, 2). This indicates that the DH technique was successful in the generation of homozygous introgression lines although at a low rate. A higher percentage (75%) of the DH lines produced carried a *T. urartu* segment in this work as compared to the wheat-*Am. muticum* DH lines where only 31% of the DH lines generated carried an *Am. muticum* introgression (King et al., 2019). However, it is difficult to assess the actual level of introgression transmission in these *T. urartu* lines as the original BC_3_ plants were not genotyped as the marker systems for the identification and characterization of the introgressions were still under development at the time of production and hence not available. Once homozygous, each introgression was stably inherited in every DH line. The generation of these DH lines accelerated the production of stable homozygous introgression lines which would have otherwise taken a number of rounds of self-fertilisation to remove heterozygosity. In addition, because the chromosome-specific KASP markers were not then available, progeny testing would have been needed in order to select lines homozygous for a segment, adding considerably to the logistics and costs of the development of the introgression lines. Due to the technical requirements of DH production and the availability of the chromosome-specific KASP markers, all lines currently in development at the WRC are being generated via a backcross and selfing programme.

### Development of Chromosome-Specific KASP Markers for *T. urartu*

Thirty three new KASP markers were also developed in this work to add to the 118 reported previously (Grewal et al., 2020a) that are polymorphic between bread wheat and *T. urartu*. This set makes up only 9% of the markers tested in this work as the SNPs used for KASP assay conversion were identified from a variety of wild relatives and wheat accessions, as described in the methods. All KASP assays designed were tested on the DH lines generated in this work to identify as many KASP markers as possible for *T. urartu*. The other 301 KASP markers also developed in this work are polymorphic between wheat and a number of other wild relative species including (but not limited to) *Am. muticum*, *Th. bessarabicum* and *T. timopheevii* (data not shown). This is also a valuable set of KASP markers that can be used in other wheat wild relative breeding programmes.

It should be noted that a majority of the KASP markers (∼70%) in each linkage group, used for genotyping the DH lines in this work, were specific to the A-genome chromosomes (Table 3) and were well distributed across all seven A-genome chromosomes. This is due to the strategy of having subgenome-specific SNPs. *T. urartu* is the donor of the A-genome and therefore it is likely that sequences that would result in such SNPs with *T. urartu* would be present more frequently on the A-genome of wheat than the B and D genomes (on average there are between 5 and 7 markers on these two genomes in each linkage group). The current KASP marker set was able to detect most of the linkage groups that were detected in the DH lines by the Axiom array with the exception of a few lines where the Axiom array found the presence of a small segment from linkage group 5 of *T. urartu* but the KASP markers did not (Table 2). This indicates that more markers need to be developed to fill in any potential gaps in linkage group 5. However, the conversion of array SNPs into chromosome-specific KASP markers able to distinguish between heterozygous and homozygous individuals in polyploid species such as bread wheat, is complex and time-consuming (Makhoul et al., 2020). We are therefore currently developing strategies to identify single-copy regions in the wheat genome in order to generate more chromosome-specific SNPs between *T. urartu* and the B and D genome chromosomes of wheat. The use of single-copy regions to develop chromosome-specific markers could bias genotyping toward genome regions without strong homoeology, which might later make it difficult to find tightly linked markers for a trait of interest if the candidate genes were found to be in highly homoeologous regions. However, the objective of this work was firstly to introgress as many wild-relative segments into bread wheat as possible and secondly to develop a suitable genotyping platform that would allow effective marker-assisted breeding. When a wild relative introgression is identified using KASP markers, the distribution of any lines carrying that segment will be accompanied by information on the markers that can be used to track the segment in any crosses with locally adapted material.

### Identifying the Size and the Recipient Wheat Genome of *T. urartu* Introgressions in DH Lines

The KASP markers allowed the identification of 65 unique *T. urartu* segments in the DH lines generated (Figure 1) and also gave an indication of the wheat chromosomes with which recombination had occurred. These 65 unique introgressions were contributed by 19 out of the 20 BC_3_ plants that were used for DH production (1 plant produced DH lines without any segments), averaging 3.4 introgressions from every donor BC_3_ plant. Some of the DH lines retained up to five introgressions potentially indicating a much higher number in the BC_1_ parent. These relatively large numbers of introgressions, even at the BC_3_ stage, are not surprising as *T. urartu* is the A-genome donor of bread wheat and homologous recombination, with the A-genome chromosomes of wheat was expected to occur at a high frequency in the F_1_ gametes as pairing between the chromosomes of *T. urartu* and the A-genome chromosomes of wheat has previously been shown to occur at a high frequency (Chapman et al., 1976; Dvořák, 1976). A majority of the introgressions (83%) were found to be with the A-genome of wheat (Table 2) as indicated by the homozygous wild relative calls for the A-genome specific KASP markers that were present within introgressions.

Due to the high similarity between the A-genome sequence of wheat and *T. urartu*, it was not possible to perform GISH analysis to observe *T. urartu* segments that had recombined with A-genome chromosomes in wheat. However, where homoeologous recombination had taken place between the *T. urartu* chromosomes and the wheat B or D genome chromosomes, it was possible to validate the genotyping using GISH analysis (Figure 2). The smallest segments that can currently be detected with GISH are about 18 Mbp and thus only the D-genome introgressions were large enough to visualise with GISH, where the markers had indicated small genomic regions of the wheat D genome chromosomes were present in recombinant chromosomes (Figure 2). This is due to the chromosome-specificity of the KASP markers which is able to firstly distinguish heterozygous from homozygous lines and secondly, in homozygous lines, to identify which wheat chromosomes had recombined with the *T. urartu* introgressions.

Only one segment from Chr 4A^u^ of *T. urartu* was found in the DH lines in this work and furthermore, it had introgressed with the Chr 4D of wheat. This is probably due to the rearrangement of Chr 4A of bread wheat as compared to Chr 4A^u^. Even though both species have the 4AL/5AL translocation as this had occurred in the diploid ancestor before the formation of hexaploid wheat (King et al., 1994; Devos et al., 1995), Chr 4A of wheat underwent a further paracentric and pericentric inversion (Devos et al., 1995; Dvořák et al., 2018) which makes homoeologous recombination between Chr 4A^u^ and Chr 4A difficult during meiosis as has been previously reported (Grewal et al., 2018a). Chr 4A^u^ and Chr 4D show greater synteny in the short arm region than Chr 4A^u^ and Chr 4A where introgression 4A.1 recombined with Chr 4D.

Multi-colour GISH analysis on 75 lines allowed the identification of the number of chromosomes in each line (Supplementary Table 1). While GISH can only indicate how many chromosomes are present per subgenome, the markers were able to identify where a pair of wheat chromosomes were missing. For example, in lines 324, 325, 327, and 329 all four chromosome 2D KASP markers gave a null call indicating that the pair of 2D chromosomes was missing in these lines. Similarly, genotyping with chromosome specific markers identified that DH lines 265 and 271 were missing a pair of 1D and 5D chromosomes, respectively. However, these markers are unable to indicate when only one of a pair of wheat chromosomes is missing.

All wheat-*T. urartu* DH lines have been deposited at the Germplasm Resource Unit (GRU) at John Innes Centre and are available to order via their website https://www.seedstor.ac.uk/ subject to the terms of the Material Transfer Agreement.

## Data Availability Statement

The datasets presented in this study can be found in online repositories. The names of the repository/repositories and accession number(s) can be found in the article/Supplementary Material.

## Author Contributions

SG, CY, SA, DS, SH-E, IK, and JK contributed to the backcross population creation. CN and AS contributed to the DH production. CY, DS, and SA contributed to the DNA extractions. AB contributed to the array genotyping. SA and DS contributed to the KASP genotyping. VG and CY contributed to the GISH analysis. SG contributed to the data analysis. IK, JK, and SG contributed to the conceptualization. SG, IK, and JK contributed to the manuscript writing. IK and JK contributed to the funding acquisition. All authors have read and agreed to the published version of the manuscript.

## Conflict of Interest

CN and AS were employed by Limagrain UK Limited at the time this work was carried out. The remaining authors declare that the research was conducted in the absence of any commercial or financial relationships that could be construed as a potential conflict of interest.

## Footnotes

1 www.biosearchtech.com/

2 https://www.cerealsdb.uk.net/
